# Critical perspectives on user involvement

**Published:** 2013-09-25

**Authors:** Margit Mayr

**Affiliations:** School of Applied Sciences Health/Social Sciences, University of Applied Sciences, Upper Austria, Austria

In the UK, user involvement became an official policy through the health and social care system. But is this concept really established and accepted by all the parties concerned? Barnes and Cotterell deal with this question in a very inspiring way. With the collection ‘Critical Perspectives on User Involvement’, the editors assemble the knowledge and experience from more than 30 contributors from the scientific field, professionals as well as from the service users themselves.

The book presents a selection of 18 separately authored texts, structured along three main parts: movements, action within services and reflection on user involvement in research. From the very beginning, the editors highlight the diverse perspectives and understandings which are reflected in a broad variety of interpretations of what user involvement means. Due to this, they provide their own definition and span an umbrella over the numerous perspectives by pointing out that differences do exist and focusing on what we can learn from them. Considering the challenge in reflecting various views in a structured way, they have succeeded in presenting a coherent concept by providing a short introduction at the beginning and a subsequent conclusion at the end of each part when they highlight questions that arise from the contributions. Compared to many other collections, this volume offers well-structured information and, where possible, cross-sectional and topical connections.

The editors start with a brief insight into the history of user involvement. This retrospection is important because it gives a pulse on how user movements and patient organisations have been established and are presently integrated in the health and social care systems. But what country are the contributors talking about? This information should come first when a collection refers to the health care system of a certain nation. With five sections, part one offers a partial view about the development of user movements and provides an idea about the motivation and experience of service users who have taken action. The contributors explore different ways in which service users have come together in order to create change. This insight is highly instructive for health care professionals and researchers to enable them to develop a better understanding of this situation. For service users, this part can be a source of encouragement, because the authors point out the power of user movements in different ways. As in part one, the second part is also structured in five sections, where user involvement in services is discussed. This section looks at initiatives that aim to enable service users to work with service providers in achieving change. In my opinion, section seven, which outlines the changing patterns of the service user involvement, is quite interesting, in particular for researchers because it provides a sense of how and why changes happen. The last part establishes reflection on user involvement in research. Barnes and Cotterell begin with a short insight into the history of development of user involvement in research, which finally has led to a well-developed infrastructure of *Patient and Public Involvement* in health and social care research. With eight sections, this chapter includes the most contributions and reflects the variety of user involvement in research in terms of the diversity of the user groups such as young mothers, young people, projects through partnership and older people. Questions such as whether the service user involvement is in danger of failing, what the impact of public involvement is and, in particular, how involvement can be measured are also examined here. Due to the fact that user involvement in research can be expected to gain in importance, as both research funds and user groups stress the relevance of user involvement, this selection of contributions provides considerably important information. With their summing up, the editors reflect on the issue from their very personal point of view. They also provide a short conclusion, but do not draw a final result over the broad range of aspects highlighted in this volume.

User involvement is an important aspect for health and social care systems and has become increasingly associated with integrated care. The particular composition of the contributors makes this volume very valuable for researchers and students in the field of integrated care. It is a collection of different voices, expressed in distinct ways and linguistic styles. While in general it is the editors’ task to draw a common thread throughout such anthologies, this collection is an exception. This is due to the fact that in this case, it is of utmost importance that readers are made aware that this topic implies the involvement of users’ voices and that the voices of those concerned are usually expressed differently from those of scientists or professionals. To do otherwise might alter the core statement of the individual parts.

With this collection, the editors published a necessary book that reflects various perspectives and constitutes a useful basis for further research on this topic. It can be recommended as a very useful tool to scientists and students as well as service users and providers.

